# Growth performance, gut microbiota composition, health and welfare of European sea bass (*Dicentrarchus labrax*) fed an environmentally and economically sustainable low marine protein diet in sea cages

**DOI:** 10.1038/s41598-023-48533-3

**Published:** 2023-12-02

**Authors:** Sébastien Alfonso, Elena Mente, Eleonora Fiocchi, Amedeo Manfrin, Arkadios Dimitroglou, Leonidas Papaharisis, Dimitris Barkas, Lola Toomey, Marilena Boscarato, Carmen Losasso, Arianna Peruzzo, Annalisa Stefani, Walter Zupa, Maria Teresa Spedicato, Ioannis Nengas, Giuseppe Lembo, Pierluigi Carbonara

**Affiliations:** 1 Fondazione COISPA ETS, 70126 Bari, Italy; 2 Laboratory of Ichthyology-Culture and Pathology of Aquatic Animals, School of Veterinary Medicine, Aristotle University of Thessaloniki, 54124 Thessaloniki, Greece; 3 National Reference Laboratory for Fish, Mollusc and Crustacean Diseases, Istituto Zooprofilattico Sperimentale delle Venezie, 35020 Legnaro, Italy; 4 Laboratory of Applied Hydrobiology, Department of Animal Science, Agricultural University of Athens, 11855 Athens, Greece; 5 Department of Research and Development, AVRAMAR S.A., 19002 Paiania, Greece; 6 Laboratory of Microbial Ecology and Genomics, Istituto Zooprofilattico Sperimentale delle Venezie, 35020 Legnaro, Italy; 7 Laboratory Medicine Service, Istituto Zooprofilattico Sperimentale delle Venezie, 35020 Legnaro, Italy; 8 Hellenic Centre for Marine Research (HCMR), Institute of Marine Biology, Biotechnology and Aquaculture (IMBBC), 19013 Anavyssos, Greece

**Keywords:** Animal physiology, Marine biology, Ecophysiology

## Abstract

The large use of fish meal/fish oil in carnivorous fish feeds is the main concern regarding environmental sustainability of aquaculture. Here, we evaluated the effects of an innovative diet, designed to be (1) environmentally sustainable by lowering the marine protein content while being (2) cost effective by using sustainable alternative raw materials with acceptable cost and produced on an industrial scale, on growth performance, gut microbiota composition, health and welfare of European sea bass (*Dicentrarchus labrax*), a key species of the Mediterranean marine aquaculture, reared in sea cages. Results show that the specific growth rate of fish fed the low marine protein diet was significantly lower than those fed conventional diet (0.67% vs 0.69%). Fatty acid profile of fillets from fish fed a low marine protein diet presented significant lower n-6 and higher n-3 content when compared to conventional ones. Then, a significant increase in the abundance of *Vibrio* and reduction of *Photobacterium* were found in the gut of fish fed with the low marine protein diet but effects on sea bass health needs further investigation. Finally, no major health and welfare alterations for fish fed the low marine protein diet were observed, combined with a potential slight benefit related to humoral immunity. Overall, these results suggest that despite the low marine protein diet moderately affects growth performance, it nevertheless may enhance environmental and economic sustainability of the sea bass aquaculture.

## Introduction

Fish production from aquaculture has greatly expanded during the last decades to face the world’s diminishing natural wild resources combined with the increasing demand for fish products^1^. In 2020, the global aquaculture production attained a record of 122.6 million tonnes^1^, being the fastest-growing food-producing sector in the world. The concerns about environmental sustainability and animal welfare in aquaculture were raised more than 20 years ago and are still increasing nowadays^2–4^. The main concerns regarding environment sustainability of aquaculture are related to the use of fish meal (FM) and oil (FO) for inclusion in aquafeeds in order to satisfy the essential nutritional requirements of marine fish in proteins and omega-3 fatty acids. These result in a high pressure on marine ecosystems since fish stocks used in aquafeeds are overall facing overfishing^1,4,5^.

Research on novel feed ingredients as replacement of FM and FO, while ensuring fish nutritional requirements, has been extensively conducted during the last years. In this way, plants, macroalgae/microalgae or insect meals have been, among other sources, considered as alternate sources of lipids and proteins for aquafeeds^5–11^. Plant-based ingredients have been particularly considered as FO and FM substitutes in feeds of various farmed fish species^6^, such as European sea bass (*Dicentrarchus labrax*)^9,12^. They were yet found to contain many biologically active antinutritional factors that can have negative effects on the feed intake, digestion and/or absorption of nutrients, as well on fish health and welfare, requiring further research on alternate substitutes^6,12,13^. Biotechnology has recently played a significant role on the improvement of nutritional value of animal feeds, by producing innovative feed ingredients and additives, originating for instance from single cells proteins (e.g. yeasts, microalgae), ensuring sustainable production performance^14,15^. In addition to sustainability questions related to the diet, which have been partially addressed in recent years by testing more sustainable raw materials (e.g. soybean-derived products, insect meal or microalgae) to reduce the final FO/FM content in current feeds^7,11,16^, important considerations for the feed industry are still the availability and cost-effectiveness of the raw materials used^11,17,18^. Indeed, even if raw materials used are more sustainable but the feed it is too much expensive due to the unavailability of some raw materials at the industrial scale, the industry may be reluctant to use it. Therefore, it is of primary interest to formulate environmentally sustainable diets that are also cost-effective (i.e., available on the market at acceptable cost) for practical use within the aquaculture industry sector^11^.

Finally, in order to evaluate the potential of using an innovative diet, one important aspect to consider, on top of growth performance, is the effect on health and welfare of the farmed fish species^6^. Indeed, in addition to environmental sustainability, fish health and welfare are a topic of great concern for consumers, producers and regulatory authorities^2,19^. It is overall accepted that fish in good health and welfare state will grow better^2^. Briefly, most of the animal welfare definitions are linked to biological functions and/or feelings^20^. On one hand, the welfare correlates with the physiological state of an organism, including blood stress indicators (e.g., cortisol, glucose, lactate). On the other hand, the welfare could be also linked with avoiding negative experiences (e.g., pain, fear, hunger)^21^. Recently, research towards health and welfare evaluation has greatly expanded across farmed fish species, requiring an overall global assessment from molecular and behavioral endpoints to biological performances^22,23^. As mentioned above, over the past 20 years, many studies have been conducted to evaluate health and welfare of fish in response to the substitution of marine proteins content in feeds^8,9,17,24^, but the ones monitoring fish under real farming conditions in sea cages have been scarce^17^, especially for Mediterranean species such as European sea bass. In the context of precision fish farming, real-time monitoring technologies appear as an interesting tool to assess health and welfare of farmed fish^25,26^. Indeed, electronic sensors evaluating fish physiological indicators, such as acceleration, heart beat rate or metabolic rate, were reported as efficiently continuous remotely monitoring fish health and welfare tools, including to monitor the fish response to acute or chronic stressors commonly observed in aquaculture environments^26–28^.

In this study, we evaluated the effects of using an environmentally and economically sustainable diet on the growth performance, health and welfare of European sea bass, a key species of the European marine aquaculture^29^, in real farming conditions (sea cages). In addition, the diet formulated in this study was designed to be (1) more environmentally sustainable by lowering the inclusion of marine protein and replacing by plant-based raw materials while ensuring the fish nutritional requirements are fulfilled and (2) economically acceptable for farmers by using alternative raw materials that were produced on an industrial scale (and therefore available in sufficient quantities the whole year round). In this study, health and welfare were evaluated using several physiological indicators, including molecular indicator (*Hsp70*) as well as hematological and biochemical blood parameters linked to stress, immunity, health and welfare (e.g., cortisol, glucose, lactate, total proteins and lysozyme concentrations). Second, some fish were implanted with accelerometer tag for continuous monitoring of acceleration, as an indicator of energy expenditure^30,31^. Finally, the gut content was sampled at the end of the experiment to evaluate intestinal microbiota composition to assess potential effects of the low marine protein diet. Overall, this study provides a wide assessment of the effects of using a more environmentally and economically sustainable diet in real farming conditions on sea bass nutritional physiology, in response to the environmental and economic sustainability challenges of the European aquaculture sector.

## Methods

### Ethical statement

Avramar S.A. research facilities are certified and have obtained the codes for the rearing and use of fish for scientific purposes (EL04-BIOexp-01). All procedures have been approved (protocol 98/24889) by the Departmental Animal Care Committee following the Three Rs principle, in accordance with Greek (PD 56/2013) and EU (Directive 2010/63) legislation on the protection of animals used for scientific purposes. Additionally, all experimental procedures were performed under the supervision of FELASA—*Federation of European Laboratory Animal Science Associations* accredited researchers. The study was carried out in accordance with the ARRIVE guidelines.

### Fish, feeding and experimental protocol

European sea bass were originated from the AVRAMAR inland hatchery (Chalkida, Greece) hatched on October 2019, year class (YC) 2019. Fish were offspring of pre-selected broodstock from AVRAMAR breeding program, consisting of 88 families (crossings). Fish were reared at the hatchery under stable conditions (mean temperature 18.2 °C, salinity 30 PSU, oxygen > 8 mg/l) until they were transferred to sea cages at the AVRAMAR farm located in Palairos (Greece) in August 2020. The experimental set up included 6 net cages (Fig. S1; 4 × 4 × 6 m), each containing approximately 2200 fish (25 fish from each family) individually pit tagged intraperitoneal with a unique radio frequency identification (RFID) tag (Biomark, Idaho, USA).

Two diets were tested in triplicate cages, i.e., the conventional diet (based on a commercial/conventional standard on-growing feed) and the environmentally and economically sustainable commercial diet with low marine protein content, formulated with economically sound raw materials easily available for the industry at the industrial scale in sufficient quantities the whole year round, while containing fishmeal replacers such as plant ingredients and yeast NuPro (Alltech Inc., Nicholasville, USA). For simplification, the commercial conventional diet and the environmentally and economically sustainable diet are latter called “conventional diet” and “low marine protein diet” respectively. The low marine protein diet was nutritionally balanced using other ingredients commonly used in aquafeeds as well as macro and micronutrients. Fat level was 2% lower in the low marine protein diet. Eicosapentaenoic acid (EPA) and docosahexaenoic acid (DHA) levels were, however, higher in the low marine protein diet since it has been observed in production practices that higher values of these fatty acids in low marine protein diets can compensate and potentially promote performance. The formulation of the diets and the chemical composition are shown in Table 1. In order to equalize the nutritional value of the two feeds, extra quantities of amino acids were added in the low marine protein diet. The fillet composition was determined according to AOAC (Association of Official Analytical Chemists) method. The amino acid profiles of the two diets were analyzed according to protocol described elsewhere^32^, and available in Table S1. The feed (as pellets) was produced by extrusion following common practices and procedures. During the experiment, feeds were stored in a cool place in line with the guidelines of the aquafeed company that produced them (AVRAMAR). During the trial, fish were fed ad libitum two to four times a day depending on sea temperature and fish size. Water temperature and dissolved oxygen levels were recorded daily. During the experimental period, the temperature was 20.4 ± 3.9 °C, ranging from a minimum of 15.2 °C and a maximum of 28 °C, while oxygen concentration averaged 7.2 ± 0.7 mg/L.

**Table 1:** Formulation and proximate composition of the experimental diets (% as fed).

Ingredients	Conventional feed	Low marine protein feed
Fish meal	20.00	12.00
Soya protein concentrate	4.80	18.40
Soya bean meal	19.50	
Plant premix	5.70	
Fermented soya		7.50
Sunflower meal	7.20	12.00
Corn gluten	18.80	17.10
Wheat	8.20	7.20
Fish oil	4.10	9.50
Salmon oil	10.30	5.50
Yeast protein		7.50
Calcium carbonate		0.50
Monocalcium phosphate	0.40	0.90
Vitamins and minerals	0.70	1.00
Methionine	0.10	0.05
Lysine	0.20	0.20
Taurine		0.05
Choline		0.10
Phospholipids		0.50
Nutrients (%)		
Moisture	8.00	7.80
Proteins	44.60	44.10
Fats	19.60	17.10
Fibers	2.80	2.90
Ash	7.10	7.20
GE (MJ/kg)	21.6	21.3
DE FSH (MJ/kg)	18.6	18.3

*GE* gross energy, *DE* digestible energy.

### Growth measurements

Fish standard length (cm) and weight (g) were recorded individually at five sampling times (i.e., T0, T1, T2, T3 and T4) over the experimental duration, on May 2020, August 2020, October 2020, May 2021 and July 2021. For practical reasons, the sampling of blood and organs corresponding to T3 and T4 were carried out in different days than the ones of the length and weight measures, corresponding to the 24-25th of April 2021 and the 06–07 of July 2021 for T3 and T4 respectively. Mortalities and feed consumption per cage were daily recorded for each cage. Specific growth rate (SGR), feed conversion ratio (FCR) and condition factor (K) were calculated per replicate according to the following formulas:

$$ \begin{aligned}{} &{\text{SGR} (\%/\text{ d)}} = {1}00 \times \left( {{\text{ln}}_{{{\text{Wfinal}}}} - {\text{ln}}_{{{\text{Winitial}}}} } \right)/{\text{d}}; \hfill \\ &{\text{FCR}} = {\text{FI }}/{\text{W}}; \hfill \\& {\text{K}} = {1}00 \times \, \left( {{\text{W}}/{\text{SL}}^{{3}} } \right). \hfill \\ \end{aligned} $$

where W_final_ is the final mean weight, W_initial_ is the initial mean weight per replicate, d is the duration of feeding (days), FI is the feed intake (g), W is the mean weight gain (g) and SL is the mean standard length (cm).

### Blood, organs, fillet and gut content sampling

At T3 and T4, a subsample of fish (n = 6 per cage at T3; i.e., n = 18 per diet and n = 6–7 per cage at T4; i.e., n = 20 per diet) was randomly selected for blood and organ sampling. Water temperature at T3 and T4 was 16 °C and 24 °C respectively. At each sampling point, fish were gently caught from cages and bathed into anesthetics (clove oil, 50 ppm) for 2–3 min before proceeding to blood sampling. The blood samples were taken from the caudal vein using a heparinized syringe, and were used to assess the levels of the different physiological indicators of fish health and welfare as described in the next section. Moreover, following blood sampling, fish were euthanized using overdose of clove oil and 100 mg of spleen, kidney, gill, liver, and brain were collected for quantitative real-time PCR (qPCR) analysis. Each sample was stored in a tube containing 1 mL of RNAlater (QIAGEN), maintained for 48 h at + 4 °C, and then stored at – 80 °C until further processing.

At the end of the experiment (T4), the fillet of a subsample of fish (n = 3 per cage; i.e., n = 9 per diet) was collected to evaluate its proximate composition (Moisture, protein, fat and ash) and its fatty acid profile. The fatty acid profiles were determined by gas chromatography^33^. The whole gut content of fish (n = 10 per cage; i.e., n = 30 per diet) was obtained by stripping, and the faeces were stored in RNA Later until further analysis.

### Quantification of hematological and biochemical parameters

The hematocrit was determined using a heparinized micro-hematocrit tube filled with blood directly from the syringe needle, which was then centrifuged at 15,000×*g* for 3 min and immediately read. Hematocrit was expressed as the red blood cell percentage of the entire blood volume. The red blood cell count (RBCC) was carried out in a Bürker counting chamber under a light microscope (Nikon 400E, Japan), and hemoglobin was measured using a commercial kit (H7379; Sigma, USA). Triplicate was used by fish for quantifying the hematocrit, hemoglobin concentration and RBCC. The remaining blood was transferred in a tube with K3EDTA (VACUMED, Torreglia, Italy) and was centrifuged at 15,000×*g* for 3 min to obtain plasma samples, which were stored at − 20 °C until further analysis. Plasma cortisol was measured using a commercial competitive electrochemiluminescent immunoassay kit (Elecsys Cortisol II Gen, Roche Diagnostics) in automatic analyzer Cobas E601 (Roche Diagnostics GmbH, Mannheim, Germany) following the manufacturer’s instructions. Plasma glucose, lactate and total protein concentrations were measured using commercial kits (Lactate Gen.2, Glucose HK Gen.3 and Total Protein Gen.2 respectively; Roche Diagnostics) following manufacturer instructions in Biochemical analyzer Cobas C501 (Roche Diagnostics). Plasma adrenalin and noradrenalin concentrations were measured using the CatCombi ELISA kits following manufacturer instructions (IBL international). The optical density of the samples and standards was measured at 405 nm in the microplate spectrophotometer Elx808 (BioTek, USA). Plasma lysozyme concentrations were measured using a turbidimetric assay modified for a microplate reader^34^. Total serum IgM levels were analyzed using the enzyme-linked immunosorbent assay (ELISA) kit (BT LAB, China) following the manufacturer’s instructions.

Electrophoresis for measuring the protein fractions content in plasma (albumin, alpha1, alpha2, beta1, beta2 and gamma) was carried out using commercial kit Hydragel PROTEIN(E) applied to the multiparametric instrument for electrophoresis Hydrasys Lc (Sebia, Bagno A Ripoli, Italy) at pH of 9.2 ± 0.1. Electrophoresis was performed on agarose gel; at the end of the electrophoretic run, the gel is dried and stained with amido Schwarz and the separated proteins appeared as bands of different intensities. The complete proteinogram was obtained following the acquisition of the gel with a scanner connected to the software Phoresis (Sebia).

### Quantification of hsp70 using qPCR

Quantification of *Hsp70* was carried out using qPCR according to the method developed in Fiocchi et al.^35^. Briefly, for each sample, the different organs were pulled together before being treated with TissueLyser II (QIAGEN, Hilden, Germany) for 1 min at 15 Hz. RNA was extracted from 30 mg of each sample by RNeasy Mini Kit (QIAGEN) following the manufacturer’s instructions. All the RNA extracts were evaluated with NanoDrop Lite Spectrophotometer (ThermoFisher scientific) for the quantification and evaluation of quality (A260/A280 ratio). Extracts were standardized using RNAase-free water to obtain a final concentration of 10 ng/μL.

To obtain first-strand cDNA, 10 ng of RNA were reverse-transcripted with SuperScript II Reverse Transcriptase Kit (Invitrogen) according to manufacturer instructions. Primer set for *Hsp70* (HSP70_bass_For: 5′-TCCTGATCTTTGACCTGGGC-3′ and HSP70_bass_Rev: 5′-GGTTGTCAAAGTCTTCCCCG-3′) was obtained from previous sequencing (NCBI, Accession number MG711592.1^35^).

A plasmid was constructed, to absolutely quantify *Hsp70* gene expression, using the TA Cloning Kit (Invitrogen). This material was obtained from the sea bass sampled during another trial^35^, total RNA was extracted and transformed into cDNA using the same protocol as described above; the product was amplified with PCR end point and subsequently was sequenced and was identified through BLAST as HSP70 of sea bass. The product was inserted into *E. coli* genome, the colonies were selected, and the genetic material was extracted again, quantified, and finally diluted to create the quantification line (from 10^10^ to 10^0^).

One μL of cDNA and one μL of plasmid in triplicate were used in 25 μL PCR reactions that included SYBR Green Master Mix (QIAGEN) and 0.4 μM of forward and reverse primer. The fragment generated by primers for *Hsp70* is 176 pb. All reactions were conducted by SYBR Green technology, so after the amplification cycle, a dissociation stage was added to estimate the specificity of the products.

### Analysis of gut microbiota composition

Total DNA for microbiota analyses was extracted from samples using a commercial column-based kit (QIAmp DNA Mini Kit, QIAGEN) following the manufacturer’s instructions. The thermal lysis of the bacterial component, was carried out at 56 °C for 2 h and RNaseA (100 mg/mL) was added to each sample to guarantee a preparation free from RNA contamination. Total DNA was resuspended in 200 µL of nuclease-free water and stored at −20 °C until library preparation. Extracted DNA was used as a template for amplification of the V3-V4 hypervariable regions of the 16S rRNA gene. The 16S library was prepared according to the Illumina 16S Metagenomic sequencing Library Preparation protocol, using the primers Bact341F and Bact785R (Fwd: CCTACGGGNGGCWGCAG and Rev: GACTACHVGGGTATCTAATCC) previously described by Klindworth et al.^36^ using the Nextera XT DNA Library Prep kit (Illumina,CA, USA). The amplification check was performed by 2% TAE agarose gel electrophoresis to identify a DNA fragment accounting for 550 bp length. Libraries were checked for both concentration and quality using Qubit and 2200 TapeStation (Agilent, CA, USA), respectively. Samples were equimolarity pooled, and sequencing was performed with an Illumina MiSeq platform using a MiSeq 600V3 cartridge (600 cycles, 2 × 300 bp, paired-end reads). After sequencing, data underwent a quality control procedure using the FastQC tool (https://www.bioinformatics.babraham.ac.uk/projects/trim_galore/). The RNA-Seq raw data generated in the present study concerning the microbiota composition were deposited in the NCBI’s Short Read Archive (accession number: PRJNA955917). All subsequent steps were performed using Quantitative Insights Into Microbial Ecology 2 (QIIME2) version 2020.2 pipeline^37^. Raw sequence data was screened, trimmed, and denoised with DADA2^38^ and quality filtered. Operational taxonomic units (OTUs) were defined as sequences with at least 97% similarity with Greengenes database (version 13.8) last release May 2013^39^. The rarefaction depth was based on the lowest read depth of samples.

Alpha diversity analysis was performed on the pre-processed count table and was measured by means of the Shannon index and observed OTUs metrics. Beta diversity was evaluated with the phylogeny based Unifrac distance metric.

### Implantation ﻿of accelerometer tags and recording of swimming activity

At T3, a sub-sample of fish (n = 5 per cage; i.e., n = 15 per diet; 352.39 ± 91.48 g) was gently randomly caught from cages and anesthetized into 30 ppm of clove oil for implantation with accelerometer tag VEMCO V9A-2x (AMIRIX Systems Inc., Nova Scotia, Canada). The tag was inserted into the fish body cavity (Fig. S1b), through an incision of ~ 1.5-cm and carefully sutured as described in Alfonso et al.^30^ under anesthesia during the whole procedure. After the surgery, an antibiotic injection (sodic-ampicillin–cloxacillin; 1 mg/kg^1^) was carried out, and fish were kept undisturbed in separate oxygenated tank (i.e., n = 15 fish from each diet in 1 m^3^) for three days of recovery. After the recovery period, all tagged fish recovered well and no mortality linked to the surgical procedure was observed. A second injection of antibiotics was done the third day after surgery and tagged fish returned to their origin cage.

The tag was programmed to measure the acceleration over two axes (X and Z), excluding the Y-axes of the backward/forward movements (Fig. S1b); acceleration being a proxy of energy expenditure^30^. Accelerometer tag transmits the tag ID and the coded values corresponding to the acceleration vector with a sampling rate of 12.5 Hertz, and high-power frequency (average delay of 120 s) to an acoustic receiver Vemco VR2AR (AMIRIX Systems Inc.) that stored data before further processing. The receiver was located at a depth of 23 m fixed on the sea bottom with weight of 20 kg, and equipped with a flotation system on the top (2.5 m from receiver), ensuring the quality of signal acquisition from the tags and the covering of all six sea cages (Fig. S1a). At T4, the receiver was recovered from the sea and data were downloaded using the software VUE (AMIRIX Systems Inc.). The acceleration values obtained from the accelerometer tags ranged from 0 to 255 and were displayed in arbitrary units (AU). The values obtained were converted into acceleration using the following equation following manufacturer’s instructions: acceleration (m/s^2^) = 0.01955(x), where x is the adimensional value returned by tags.

### Statistical analysis

Statistical analysis was carried out using the R software version 4.0.4^40^ at the 95% level of significance. Data are presented as mean ± SD (standard deviation) expected if otherwise mentioned. Repeated measures ANOVA was performed for the analysis of the average fish weight for each cage in relation with the five sampling points followed by the Greenhouse–Geisser correction. In addition, independent sample T-tests were applied for the analysis of the growth data (K-factor, mortalities, FCR and SGR), and ANOVA followed by Tukey’s honest significant difference (HSD) was performed to analyze the fillet composition.

Regarding gut microbiota data, non-parametric Kruskal–Wallis test was used to compare alpha diversity between fish microbiota feed by conventional and low marine protein diet. The PERMANOVA test was used to compare the beta diversity parameters between the two diet groups. Differential abundance at Class and Genus taxonomy level was evaluated using the analysis of compositions of microbiomes with bias correction method^41^.

A generalized linear mixed model (GLMM) was used to compare the swimming activity between the two diet groups (conventional vs. low marine protein), the time of day (day- or night-time) and the interaction between diet and time of day as fixed factors and the fish ID as a random factor, using the package lme4^42^. Gamma family was used. In addition, an analysis of frequency distribution between the two diets has been carried out on the whole data set of swimming activity values recorded by tags during the experiment, merging by slot of 10 the swimming activity values (i.e., 0–10, 11–20, […], 241–250, 251–255). The statistical analysis has been carried out first by using Chi-squared test, and then by using row-wise z-tests of two proportions with p value adjusted by Bonferroni, to compare the proportion of values slot by slot between the two diets using the package rstatix^43^.

For the analysis of the physiological parameters related to fish health and welfare, a GLMM model was applied to compare each parameter between the two diet groups (conventional vs. low marine protein), the sampling time (T3 vs. T4) and interaction of diet and sampling time as fixed factors and the cage ID as a random factor. If interaction was non-significant, it was removed in the final model. GLMMs were followed by Tukey’s HSD to test differences between groups when relevant. For each model, the family was selected according to the data distribution and is available in Table S3 and Table S4.

## Results

### Growth performances and fillet composition

At the start of the experiment (T1) the initial fish weights in both dietary treatments were similar, (mean ± SD: 40.9 ± 0 g for both diet treatments, n = 3 cages; Fig S2). Among the five time points monitored, there was a significant effect of time*diet interaction on the weight (p < 0.05; Table 2). The average weights differed significantly only at the end of the experiment (T4); the final average weight of fish fed the conventional diet was significantly higher compared to the average final weight of fish fed the low marine protein diet (474.8 ± 9.6 g vs. 433.5 ± 3.6 g; p < 0.05; Fig. S2). In addition, the K-factor and total mortalities showed no significant differences (p > 0.05) between the two diet groups (Table 2). Similarly, FCR showed no significant statistically differences between the two dietary treatments for each of the time periods (p < 0.05; Table 2). Finally, SGR was different between the two diet groups only when considering the whole trial period (T1–T4) but not when using partial sampling times (i.e., T1–T2 and T1–T3) (p < 0.05; Table 2).

**Table 2:** Growth parameters (K, FCR, SGR (%)), feed quantity provided and mortality during the experimental period for the two diet groups (conventional vs. low marine protein; n = 3 cages per diet group).

Diet	K	FCR			SGR (%/day)			Mortality (n)	Mortality (%)
		T1–T2	T1–T3	T1–T4	T1–T2	T1–T3	T1–T4	T1–T4	T1–T4
Conventional diet	1.74 ± 0.01	2.03 ± 0.19	1.87 ± 0.03	2.06 ± 0.09	1.39 ± 0.04	0.74 ± 0.00	0.69 ± 0.01^a^	483 ± 29	22.03 ± 1.30
Low marine protein diet	1.76 ± 0.02	2.08 ± 0.24	1.84 ± 0.2	1.98 ± 0.17	1.35 ± 0.06	0.73 ± 0.02	0.67 ± 0.01^b^	457 ± 85	20.87 ± 3.97

Different letters in the same column indicate significant different values between diet groups (T-test; p < 0.05).

*K* condition factor, *FCR* feed conversion ratio, *SGR* specific growth rate.

Overall, the fillet composition of fish fed with the low marine protein diet did not differ statistically from fish fed with conventional diet (p > 0.05 for all; Moisture: 71.73 vs. 71.68%, protein: 19.64 vs. 19.81%, fat: 7.51 vs. 7.45% and Ash: 1.40 vs.1.40% for fish fed conventional diet vs. fish fed low marine protein diet). Fillet fatty acid profile of fish fed with the low marine protein diet showed some statistical differences from fish fed with the conventional diet (Table 3; p < 0.05). In more details, the fillets composition of fish fed with plant and yeast ingredients demonstrated overall higher total n-3 content, including for EPA and DHA, and lower n-6 content, including for arachidonic acid (ARA) (Table 3; p < 0.05).

**Table 3:** Fatty acid composition (% of total) of European sea bass fillet fed the two experimental feeds (n = 3 cages per diet group).

Fatty acids	Conventional diet (%)	Low marine protein diet (%)
14:0	2.63	3.36
14:1	0.04	0.05
15:0	0.28	0.31
16:0	16.80	17.19
16:1n-9	0.54	0.46
16:1n-7	4.28	5.57
17:0	0.29	0.27
17:1n-7	0.23	0.21
18:0	3.98	3.76
18:1n-9	31.51	26.30
18:1n-7	3.29	3.23
18:2n-6	12.22^a^	9.58^b^
18:3n-3	3.70	2.68
18:4n-3	0.81	1.41
20:0	0.24	0.21
20:1n-11	0.33	0.55
20:1n-9	3.39	4.88
20:2n-6	0.94	0.67
20:3n-6	0.16	0.12
20:4n-6 (ARA)	0.55^a^	0.48^b^
20:3n-3	0.27	0.21
20:4n-3	0.51	0.50
20:5n-3 (EPA)	3.95^a^	5.15^b^
22:0	0.09	0.08
22:1n-11	1.58	4.12
22:1n-9	0.45	0.61
22:6n-3 (DHA)	6.53^a^	7.59^b^
24:1n-9	0.43	0.46
Total		
Saturated	24.30	25.18
Monounsaturated	46.06	46.43
n-9	36.32	32.71
n-6	13.87^a^	10.84^b^
n-3	15.76^a^	17.54^b^
EPA	3.95^a^	5.15^b^
DHA	6.53^a^	7.59^b^

Different letters in the same line indicate significant differences between diet groups (ANOVA followed by Tukey’s HSD; p < 0.05).

*ARA* arachidonic acid, *EPA* eicosapentaenoic acid, *DHA* docosahexaenoic acid.

### Composition and diversity of gut microbiota

**Figure 1:**
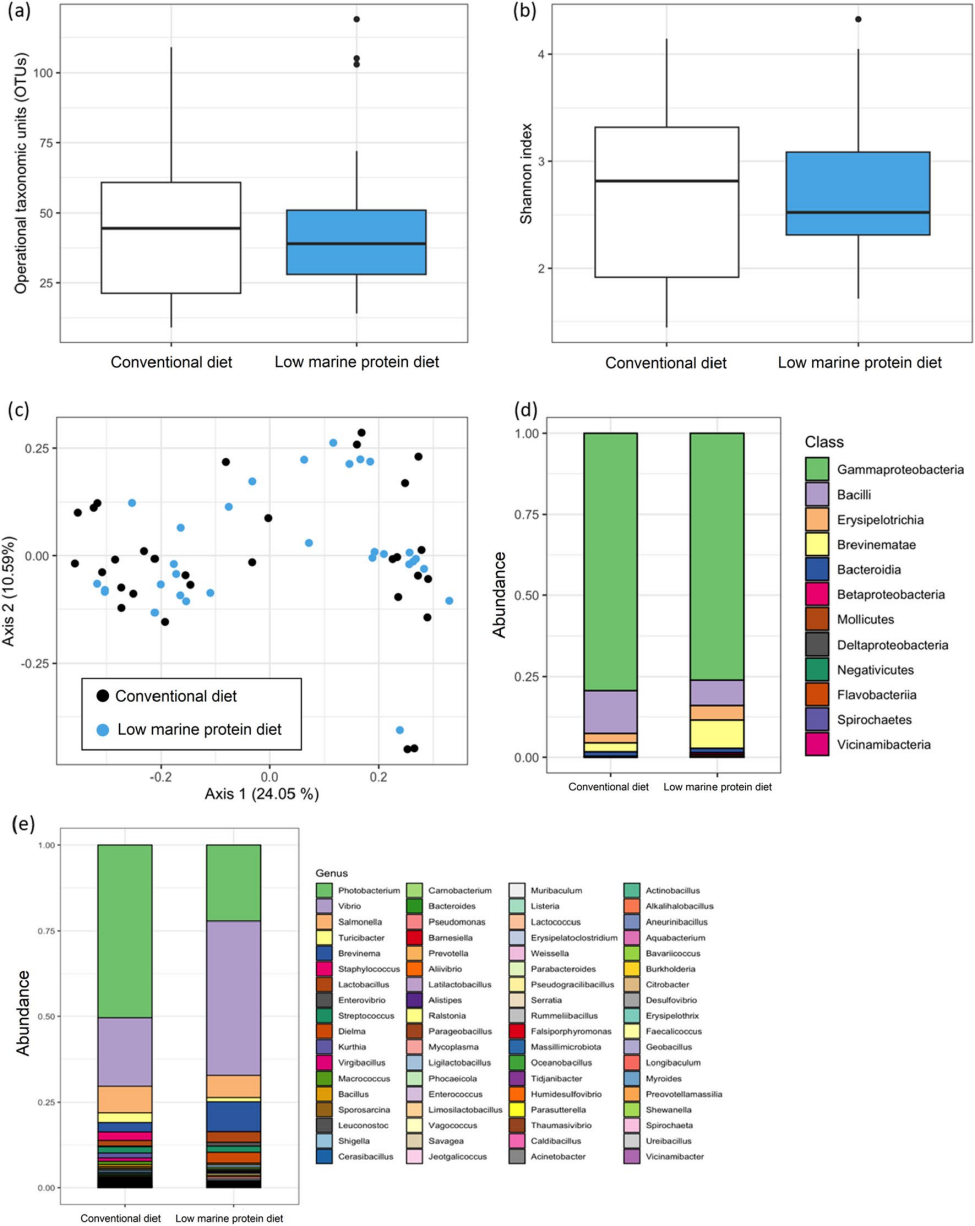
**(a)** Number of identified OTUs in the gut and **(b)** Shannon index for the two diet groups (white boxplot—conventional diet; blue boxplot—low marine protein diet). The central line of the boxplot indicates the median and the boxes the quartiles, with the whiskers covering 95% of the values. Outliers are represented by black dots. **(c)** Beta diversity analysis using Principal Coordinate Analysis (PCoA) plot based on Unifrac distance matrix calculated between the fish from the two diet groups (black circle—conventional diet; blue circle—low marine protein diet). Average abundance of bacterial taxa at the **(d)** class and **(e)** genus level, respectively, between the fish from the two diet groups (n = 30 fish per diet group).

No significant differences were found in the number of identified OTUs and in the Shannon index between the microbiota of fish fed with the low marine protein diet and those fed the conventional diet (p > 0.05; Fig. 1a, b). The same conclusions were reached for beta-diversity metrics (p > 0.05). In more details, the overall structure of the microbial community for each group of fish does not differ from each other (Fig. 1c). A total of 369 OTUs were found to be common amongst fish fed with the two different diets, while 280 OTUs and 272 OTUs were unique in fish fed with conventional and the low marine protein diet, respectively.

In addition, 90% of microorganisms in the samples of the entire dataset belonged to three classes (*Gammaproteobacteria*, *Brevinematae* and *Bacillus*) (Fig. 1d). The pairwise comparison between the two diet groups revealed the presence of two classes unevenly distributed: *Brevinematae* and *Bacillus*. The *Brevinematae* were found in a higher frequency in fish fed with the low marine protein diet than fish fed with the conventional diet; on the contrary, the class of *Bacillus* was reversely distributed among groups. Focusing at the genus level, it can be seen that in fish fed the low marine protein diet, there is an increase in OTUs belonging to the genera *Vibrio* (+ 25%) and *Brevinema* (+ 6%) and a decrease in those belonging to the genus *Photobacterium* (− 28%) (Fig. 1e). Differential abundance analysis revealed that the class of *Mollicutes* was over-represented in fish fed with the low marine protein diet. At the genus level, there were ten genera of bacteria that displayed different abundances between the two groups of fish. Among them, *Kurthia*, *Staphylococccus*, *Virgibacillus*, *Cerasibacillus*, *Prevotella* and *Vagococcus* are over-represented in fish fed with the conventional diet while *Ralstonia*, *Mycoplasma*, *Ligilactobacillus* and *Citrobacter* were over-represented in fish fed with the low marine protein diet but with difference between the two diets not exceeding 1.4%.

### Swimming activity data recorded by tag

**Figure 2:**
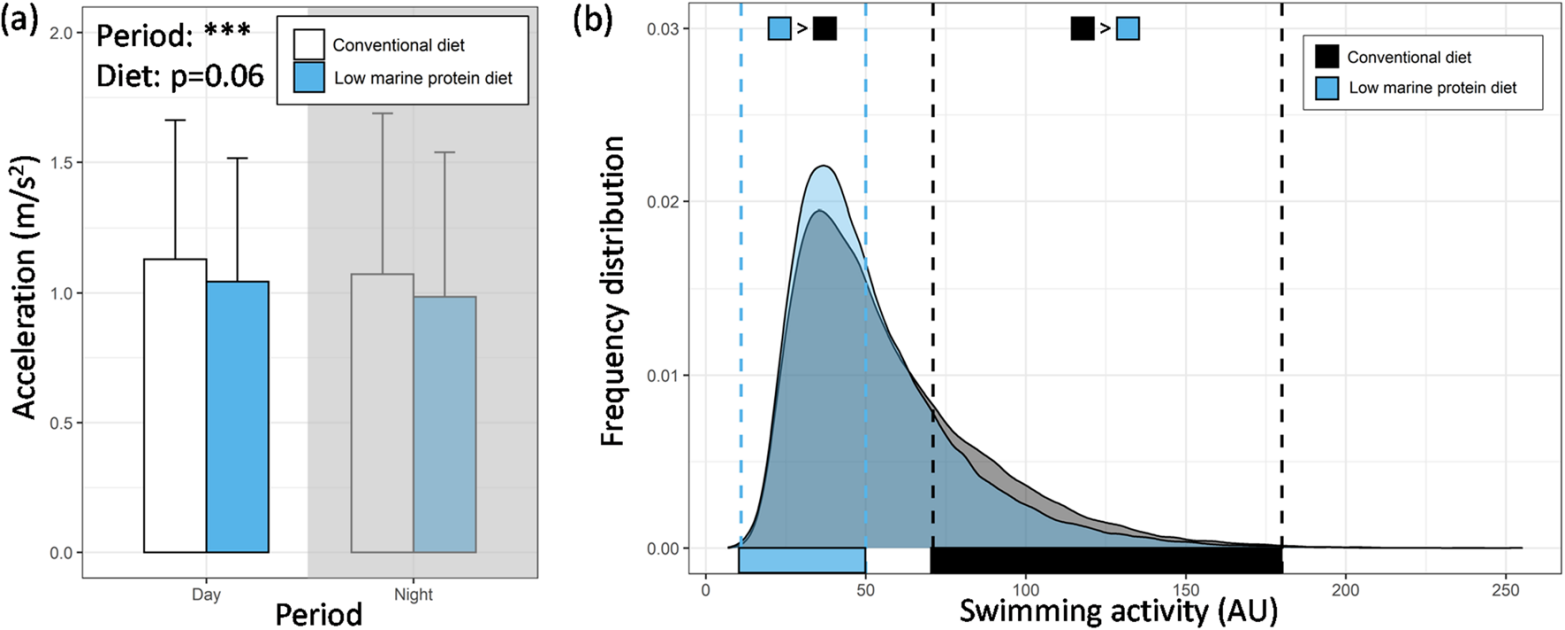
**(a)** Acceleration (mean ± SD; m/s^2^) as a function of period of the day (i.e., day and night) for conventional diet (white bar; n = 14) and the low marine protein diet (blue bar; n = 15). Grey background indicates the night period. Statistical result related to GLMM model (Table S2) is indicated on the top left, and **(b)** frequency distribution of the swimming activity values (in arbitrary units; AU) as a function of conventional (filled square) and the low marine protein diet (blue square). diets. Differences in the frequency distribution between the two diets are highlighted on the figure according to row-wise z-tests (p < 0.05).

The period of the day significantly affected the acceleration of sea bass; sea bass were more active during the daytime than nighttime regardless of diet treatment (Table S2; Fig. 2a; p < 0.001). However, the diet did not significantly affect the acceleration displayed by sea bass, even if a trend for decreased acceleration in sea bass fed by the low marine protein diet was observed (Table S2; Fig. 2a; p = 0.06). If the overall acceleration was not found different between the two dietary treatments, the distribution of swimming activity values recorded by tags did appear different between the diets (p < 0.001). In more details, a higher data frequency for low and medium swimming activity values (11 < x < 60 AU) was found in fish fed the low marine protein diet (p < 0.05; Fig. 2b). On the contrary, for values ranging from 71 to 180 AU, a higher data frequency was observed in fish fed conventional diet compared to fish fed the low marine protein diet (p < 0.05; Fig. 2b).

### Health, stress and welfare parameters

**Figure 3:**
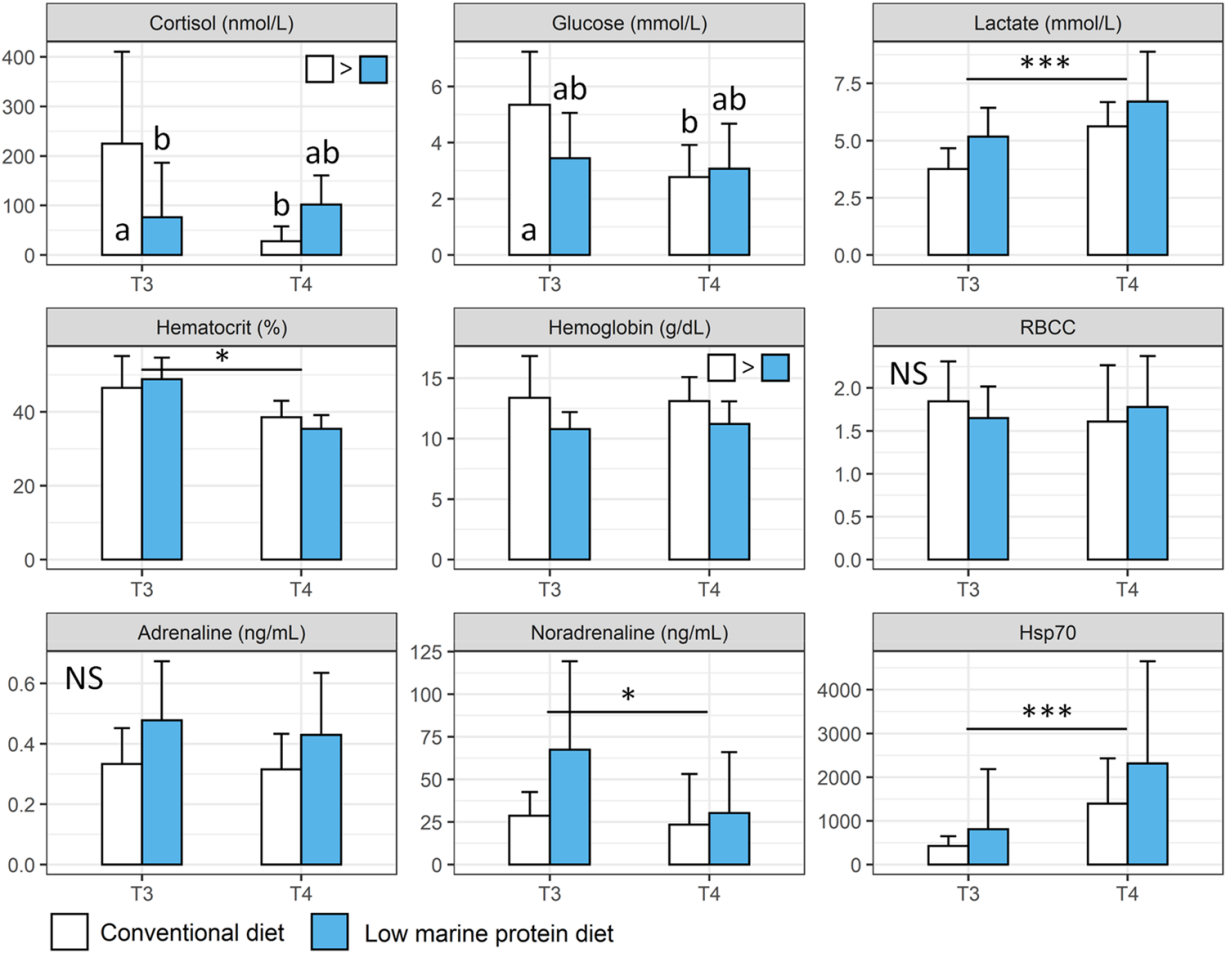
Stress, health and welfare blood physiological parameters (mean ± SD) measured at the two different sampling points (T3 and T4) in the conventional diet (white bar; n = 18–20) and the low marine protein diet (blue bar; n = 18–20) groups. Parameters monitored are cortisol (nmol/L), glucose (nmol/L), lactate (nmol/L), hematocrit (%), hemoglobin (g/dL), red blood cell count (RBCC; 106 cells/mm3), adrenaline (ng/mL), noradrenaline (ng/mL) and absolute level of Hsp70 (number of copies/µl; measured in pull of organs). Symbols indicate significant different between the two sampling times (*: p < 0.05; **: p < 0.01 and ***: p < 0.001). When an effect of diet was detected, it was indicated on the top right of each graph using colored squares. Different letters indicate statistically significant differences between the different groups (diet groups and sampling times) while NS indicates no significant difference neither between diet groups nor sampling times (GLMM followed by Tukey’s HSD post-hoc test; see details in Table S3).

Some parameters such as RBCC and adrenaline did not vary over neither the trial period nor across diet groups (Fig. 3; Table S3; p > 0.05 for both parameters and effects). Only the sampling time has a significant effect on most of the stress, health and welfare parameters monitored (i.e., lactate, hematocrit, noradrenaline and *Hsp70*; Fig. 3; Table S3; p < 0.05). Significant interaction effect between diet and sampling time was found for both cortisol and glucose (Fig. 3; Table S3; p < 0.05). Overall, fish fed conventional diet displayed greater level of cortisol and tendency for greater level of glucose than fish fed the low marine protein diet in plasma (Fig. 3; p < 0.05 and p = 0.06 for cortisol and glucose respectively). At T3, fish fed conventional diet displayed greater level of cortisol than fish fed the low marine protein diet (p < 0.05). but differences disappeared at T4 (Fig. 3). Fish fed conventional diet also displayed higher level of cortisol at T3 than T4 (Fig. 3, p < 0.05). Concerning glucose, the level was greater at T3 than T4 for fish fed conventional diet (p < 0.05) but no differences between the two diets were found within sampling times (Fig. 3; p > 0.05). Also, hemoglobin was found to be globally higher in blood of fish fed conventional diet than fish fed the low marine protein one (Fig. 3; p < 0.05).

**Figure 4:**
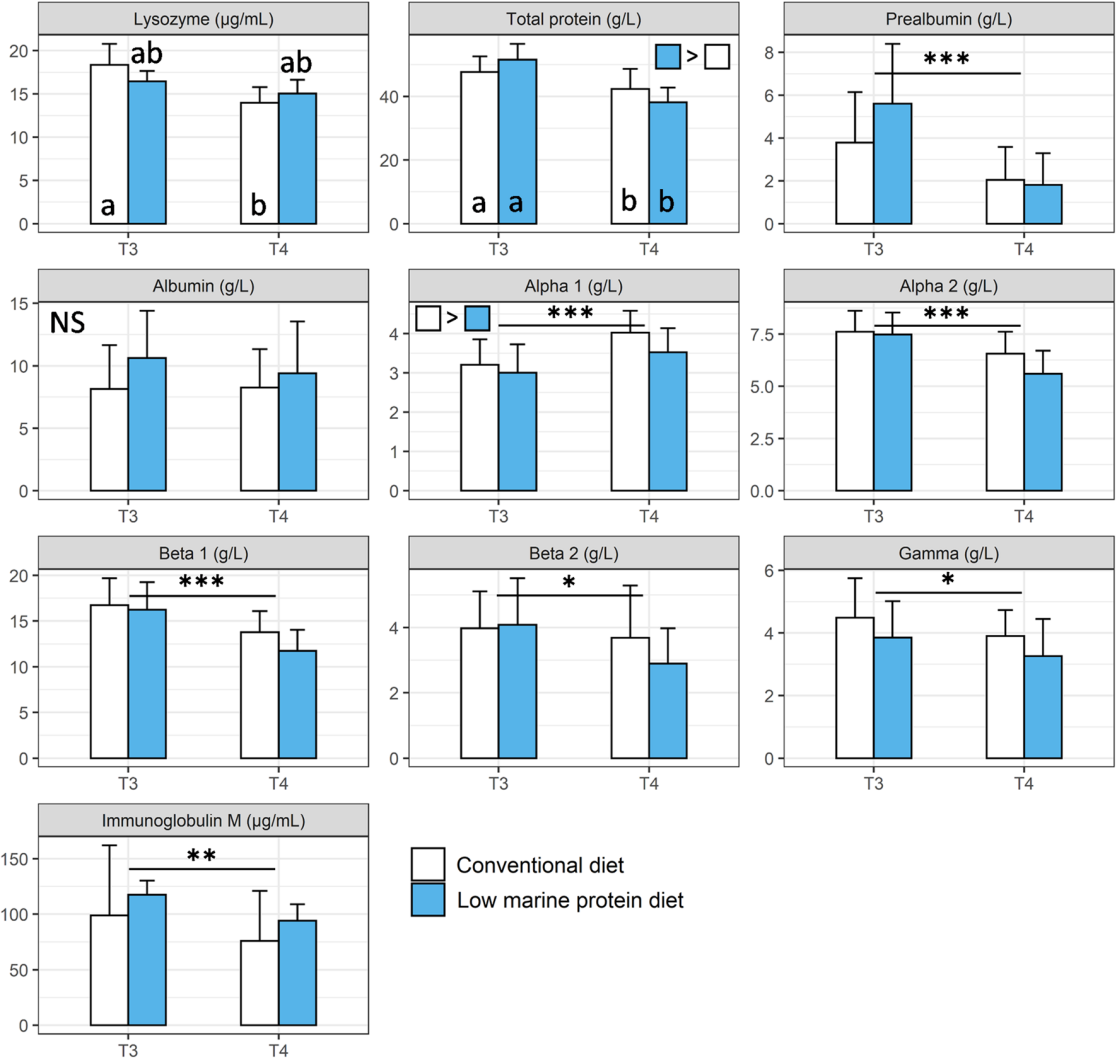
Proteins content and immune blood parameters (mean ± SD) measured at the two different sampling points (T3 and T4) in the conventional (white bar; n = 18–20) and the low marine protein diet (blue bar; n = 18–20) groups. Parameters monitored are lysozyme (µg/mL), total protein (g/L), prealbumin (g/L), albumin (g/L), alpha 1 (g/L), alpha 2 (g/L), beta 1 (g/L), beta 2 (g/L), gamma (g/L) and immunoglobulin M (µg/mL). Symbols indicate significant differences between the two sampling times (*: p < 0.05; **: p < 0.01 and ***: p < 0.001). When an effect of diet was detected, it was indicated on the top right of each graph using colored squares. Different letters indicate statistically significant differences between the different groups (diet groups and sampling times) while NS indicates no significant difference neither between diet groups nor sampling times (GLMM followed by Tukey’s HSD post-hoc test; see details in Table S4).

Total protein concentration was also globally higher in fish fed the low marine protein diet than fish fed conventional diet (p < 0.05) but no specific differences between diets were found within sampling times (Fig. 4; p > 0.05). When looking at the concentration of the different protein fractions, only the albumin concentration did not vary over time or between diets (Fig. 4; Table S4; p > 0.05 for both effects) while the sampling time has a significant effect on most of them (i.e., prealbumin, alpha1, alpha2, beta1, beta2, gamma and immunoglobulin M; Fig. 4; Table S4; p < 0.05). In addition, the concentration of alpha1 globulin was overall lower in fish fed the low marine protein diet than fish fed conventional diet (p < 0.05). Finally, a significant interaction effect between diet and sampling time was observed for lysozyme (Fig. 4; Table S4; p < 0.05). In more details, the lysozyme concentration in plasma was higher in fish fed conventional diet at T3 than T4 but no differences were observed between diets or across time for the low marine protein diet (Fig. 4; p < 0.05).

## Discussion

In this study, we evaluated the effects of an environmentally and economically sustainable diet with low marine protein content (i.e., lowered from 20 to 12% and replaced by plant and yeast proteins) on the growth performance, gut microbiota composition, health and welfare of farmed European sea bass in real farming conditions. With such partial substitution of the marine protein content by sustainable and available raw materials, the diet tested here may render the European sea bass aquaculture more sustainable and environmentally friendly while being economically sustainable for farmers. In more details, the two main requirements for formulating the diet to be economically sustainable for farmers were that (1) the alternative raw materials used must be produced on an industrial scale and therefore be available in sufficient quantities the whole year round, and that (2) the cost of the final fish feed must be acceptable from the producers. In 2020, when these feeds were tested, yeast protein and fermented soya used met the abovementioned requirements. With the prices of the raw materials that were valid at that time, the experimental feed had a 3% improved cost compared to the conventional one, while with the present prices in 2023, the experimental feed has an increased cost of 5%. However, differences of ± 5% in industrial production are still within the competitive scope which makes the experimental feed tested sustainable until now. However, the use of such environmentally and economically sustainable diet should not be at the cost of altering significantly the fish physiology, including immunity, health and growth, in order to be used in the aquaculture sector^6^.

At the end of the trial, the average final weight of fish fed with the conventional diet was 475 g while it was 434 g for the fish fed the low marine protein one, resulting in a difference of about 40 g between the two diet groups (~ 8% of the weight of fish fed conventional diet). Such difference can be filled in 10 or 20 days in the warm or in the cold period in the Mediterranean Sea, respectively. Significant differences in the final weight were due to differences in the growth rate; fish fed the low marine protein diet had slightly lower SGR than fish fed the conventional one (0.69 vs. 0.67%) when considering the whole monitoring period. In addition, there was no statistically significant difference in the FCR between the two groups although FCR was lower for the low protein marine diet (1.98 vs. 2.06). Therefore, we suggest that the growth difference at the end of the trial should be attributed to the fact that fish fed the low marine protein diet used more efficiently the feed resources and consume less feed than fish fed the conventional diet (mean of 1321 vs. 1500 kg of feed consumed per cage). Also, there were no differences in either the K or mortality, overall supporting good performances for fish fed the low marine protein diet. About mortality, we reported an overall mortality rate of approximately 20% across all diets tested in this study (22.03 ± 1.30% and 20.87 ± 3.97% for conventional diet and low marine protein diet, respectively). This is consistent with what is expected for European sea bass, which typically experiences a 15–20% mortality rate throughout the entire grow-out stage, mainly attributed to bacterial diseases (74%)^44,45^. Finally, the fillet composition of sea bass fed with the low marine protein diet did not differ with fish fed conventional diet in term of proximate composition (moisture, proteins, fat and ash). However, the fillet composition of fish fed the low marine protein diet, tested here, demonstrated higher total of n-3 content, including EPA and DHA, and lower n-6 content, including ARA, than fish fed the conventional diet, showing overall good nutritional values for fish fed the low marine protein diet. Indeed, the replacement of fish oil and fish meal by other alternate sources, such as plant derived products, in aquafeeds is often characterized by a decreased amount of nutritionally interesting long chain n-3 polyunsaturated fatty acids (PUFA), such as EPA and DHA acids, in fish fillets, increasing the n-6/n-3 ratio of final fish products^46–48^. Such observed results in the present study are due to the profile and the quantities that fish oil and salmon oil are incorporated in the low marine protein diet to increase its performance. Yet, producing fish fillets with the highest content in n-3 highly unsaturated fatty acids (HUFA) is crucial since these fatty acids were reported to have beneficial effects for human health. In this study, fillets from fish fed the low marine protein diet displayed even higher n-3 PUFA content and lower content in n-6 PUFA than fish fillets from the conventional group, suggesting an interesting nutritional profile of fillets for human consumption.

No difference was observed in alpha and beta-diversity indexes between the gut microbiota of fish fed with the low marine protein and conventional diet. A total of 369 OTUs were found to be common amongst fish fed with the two different diets, while 280 OTUs and 272 OTUs were unique in fish fed with conventional and the low marine protein diet, respectively. This means that although the diversity of microorganisms in the microbiota is similar between the fish fed the two diets, there are differences in the microorganisms found, suggesting an effect of diets in promoting some particular OTUs. Overall, 90% of microorganisms in the samples of the entire dataset belonged to three classes (*Gammaproteobacteria*, *Brevinematae* and *Bacillus*) and about 70% of microorganisms belonged to two genera (*Photobacterium* and *Vibrio*). This is consistent with meta-analysis of the gut communities of marine fish that revealed that Vibrionales bacteria (which includes the genera *Vibrio* and *Photobacterium*) accounted for 70% of sequence reads^49,50^. In European sea bass, many studies were published recently and the overall composition of gut microbiota may significantly vary from a study to another^24,51–55^, suggesting that many factors are affecting the gut microbiota composition, such as life stage, sex, season, habitat and diet^50,56,57^.

In the present study, there was an increase in OTUs belonging to the genera *Vibrio* (+ 25%) and *Brevinema* (+ 6%) and a decrease in those belonging to the genus *Photobacterium* (− 28%) in fish fed the low marine protein diet in comparison with fish fed the conventional diet. *Vibrio* is a genus belonging to the *Proteobacteria* phylum and is overall known as one of the most important bacterial genera in aquaculture mainly for its different pathogenic species (*Vibrio anguillarum*, *Vibrio salmonicida* and *Vibrio vulnificu*). Many *Vibrio* species are, however, not true pathogens, but in fact opportunistic pathogens whose virulence is accentuated under intensive aquaculture conditions^58^. It is worth mentioning that the genus also contains health-promoting species (e.g. *Vibrio alginolyticus*), and that many *Vibrio* species are acting as symbionts producing hydrolytic enzymes to assist in the breakdown of dietary components^50,59,60^. As *Vibrio*, *Photobacterium* is also a genus of the *Proteobacteria* phylum. Many *Photobacterium* bacteria act as mutualistic bacteria in the host gut for chitin digestion^50^. However, some also produce harmful enzymes such as neuraminidases (e.g., *Photobacterium damselae*)^61^, and some species may act as common pathogen for fish (e.g. pasteurellosis), also able to induce skin ulcer^51,62,63^. Interestingly, such concomitant changes (i.e. increased abundance in *Vibrio* and reduced abundance of *Photobacterium*) were previously observed in European sea bass fed with feed supplemented with algal extract (0.35%) but only reduction of *Photobacterium* abundance has been found when inclusion of algal biomass (2.5% or 5%) in feed^51^. Gonçalves et al.^51^ interpret the reduction in the abundance of *Photobacterium* as a limitant for its role as a permanent and latent member of the intestinal microbiota. Specific resistance to infection with *Photobacterium damselae subsp. piscicida* has been highlighted for gilthead sea bream (*Sparus aurata*) fed with such diet (algal biomass of 5%), but not in sea bass^64,65^. Better resistance to infection in sea bream was also linked to enhanced immune features (i.e. increased plasmatic lysozyme concentration) and growth^64^. This contrasts with our study as growth has not been enhanced and potential benefits in immune features is overall limited in fish fed the low marine protein diet (i.e., only increased in total proteins concentration). Based on the current data, it is difficult to interpret the effects of combined increased in *Vibrio* species to the reduction of *Photobacterium* species abundance in gut microbiota of fish fed the low marine protein diet, whether these changes would be positive or negative for fish health. This likely depends on specific factors related to aquaculture conditions (e.g., Vibrio virulence accentuated under intensive aquaculture conditions, probability of *P. damselae* infection).

To date, *Brevinema* has been reported twice as a member of the European sea bass gastrointestinal tract habitat in mucosa sample by Serra et al.^24^ and Torrecillas et al.^53^. It is very unlikely that *Brevinema* to be a pathogen in sea bass as its optimal growth is 30–34 °C and it typically does not grow below 25 °C. *Brevinema* has been found among the dominant bacteria in the gut of various species such as Nile tilapia (*Oreochromis niloticus*)^66^, rainbow trout (*Oncorhynchus mykiss*)^67^, Atlantic salmon (*Salmo salar*)^68^ or common carp (*Cyprinus carpio*)^69^. The ecophysiological role of *Brevinema* in teleosts remains largely unknown, but its presence in the fish gut has been related for instance to increased supply of complex/non-digestible carbohydrates such as xylooligosaccharides and galactooligosaccharides^70^ or *Spirulina* supplement^69^. These studies imply that *Brevinema* outcompete other fish gut bacteria in the utilization of complex carbohydrates provided by the supplied aquafeed. It is likely that its dominance in our experiment is due to some of the feed ingredients such as yeast protein, fermented soya and sunflower meal. Further studies in real farming conditions are needed to specifically define the effects of such variations in *Brevinema* abundance, as well as the other genera, in gut microbiota on the European sea bass health.

When looking at the blood physiological parameters investigated, no major alterations of health and welfare for fish fed the low marine protein diet were found. Variations in the concentration of parameters investigated were mostly observed between the two sampling times, which is not surprising since water parameters, such as temperature, differ between the two sampling times due to seasonal changes^71,72^. The concentration of some physiological parameters however differed between fish from the two diet groups. First, cortisol level was found overall higher in fish fed conventional diet than the low marine protein one. This could suggest higher stress level for these fish but post-hoc tests carried out indicated that cortisol was higher in fish fed conventional diet compared to the low marine protein ones only at T3. Plasma cortisol level is known to be a more reliable marker for acute than chronic stress^73^. Since the levels are similar between diet groups at T4, this could suggest that differences observed at T3 could be linked to a possible acute stress that happened before sampling. It is worth mentioning that care was taken to minimize stress-induced by handling for both fish diet groups during sampling, and that cortisol values measured at T3 for both diet groups were within the range of what can be expected for basal level in European sea bass^74,75^. Moreover, we did not measure any significant difference between the two diet groups regarding glucose concentration or in the expression of *Hsp70* protein, a reliable stress indicator in fish, including European sea bass^35^ (although some high *Hsp70* levels were measured in some samples of fish fed the low marine protein diet at both T3 and T4; see Fig. 3). Finally, we overall measured greater level of hemoglobin in fish fed conventional diet than fish fed the low marine protein diet. Typically, greater concentration of hemoglobin in blood could be translated into increased oxygen delivery to tissues, and this could explain the better growth measured in fish fed conventional diet^76^. It is, however, worth mentioning that the hemoglobin levels are within the expected range for fish fed with both diets. The difference seen in hemoglobin concentration should thus not significantly affect health of sea bass^76,77^. This is also supported by the lack of difference between the two diet groups for closely related parameters to hemoglobin, such as hematocrit and RBCC, which are also within the normal range for sea bass^76,77^.

Regarding blood proteins and immune parameters, the concentration of most parameters investigated was also found similar between the two diet groups, which is consistent with the mortality data showing no difference between the two groups. This is an interesting result because, although growth is an important parameter when designing and testing diets with new raw ingredients and formulas, immunity is a key factor for ensuring disease resistance and fish health and subsequent farming productivity^6^. Only total proteins concentration was found different between the two diet groups; with greater concentration in fish fed the low marine protein diet. Typically, total proteins level is a useful blood parameter to be measured since total proteins cover a wide range of physiological functions, including maintaining pH and osmotic pressure, transporting various metabolites and interacting with immune system^76^. The concentration of total protein is known to decrease in relation with many diseases, mainly due to decreased capacity of synthesis, reduced absorption or protein loss^78^. Here, the only difference observed in specific protein fractions between the two diet groups was regarding the alpha1 globulin fraction, being part of the acute-phase proteins^79,80^. Also, no difference was observed between the two diet groups regarding the other immune parameters investigated (e.g., lysozyme, IgM), overall suggesting that the total protein concentration differences between the two diet groups may be linked to other functions than immunity and/or that there is a slight enhancement of humoral innate immunity.

Physiological sensors represent valuable tools for fish health/welfare monitoring in aquaculture since they are providing a continuous remote monitoring of physiological parameters of interest without disturbing fish with repeated sampling events^26^. A recent review reports about 25% of mortality following fish tagging in sea cages, suggesting the sea cages as more risky environments than indoor aquaculture tanks for the use of such tags^81^. In this study, 29 of the 30 tagged fish survived over the whole experiment, representing a low mortality of 3.3% in comparison with the mean mortality observed by Macaulay et al.^81^. That could be due to our protocol in which, following the surgical procedure, tagged fish were kept three days in tanks, away from sea cages stressors (high density, competition), to ensure recovery before being reintroduced into sea cages. In addition, the implantation of tag was demonstrated to not induce stress in sea bass by monitoring different endpoints (e.g., growth, cortisol, MO_2_)^30,82^ but it is worth mentioning that some behavioral alterations (group cohesion, polarization) may be observed following tagging^83^. In this trial, we continuously monitored the acceleration of sea bass fed with the two different diets over a two-months period. Acceleration is known as a reliable proxy of energy expenditure in this species^30,31^. During the experiment, no significant difference between the two diet groups was found but a tendency for greater acceleration level in fish fed conventional diet than fish fed the low marine protein was highlighted. Evaluating the distribution data, the distribution patterns of the recorded acceleration values were different between the two dietary groups. In more details, fish fed the low marine protein diet display higher data frequency for low and medium swimming activity values (from 11 to 60 AU) while fish fed conventional diet display higher data frequency for high swimming activity values (from 71 to 180 AU). In a previous study, the activity of red and white muscle of European seabass, which is indicative of aerobic and anaerobic metabolism respectively, was monitored using electromyograms electrodes during swimming^30,31^. It was concluded that for swimming activity values < 70 AU recorded by tag, fish swimming is fueled by the aerobic metabolism while for values of ~ 71–125 AU, the fish starts to progressively consume some energy from anaerobic metabolism until 255 AU (limit of tag measurement)^30^. In the present study, the swimming of fish fed the low marine protein diet appears to be overall more fueled by the aerobic metabolism while, on the contrary, fish fed by conventional diet consume more energy from anaerobic metabolism. Use of anaerobic metabolism is typically correlated with higher lactate level in blood^84^, which has not been observed in this study. This could be explained because handling prior blood sampling may mask some differences, while the tag data were obtained from long time monitoring without disturbing fish and are hence considered more robust.

In conclusion, the inclusion of plant and yeast proteins to replace 40% of the proteins from wild fish origin in European sea bass commercial conventional diet (lowered from 20 to 12%) has been proved promising in real farming conditions in this study. Lowering the quantity of fish meal in the low marine protein diet led to the production of farmed European sea bass with fish in fish out ratio close to one. In addition, being more environmentally sustainable, the feed used in this study was developed in collaboration with the aquaculture industry to also be economically sustainable for farmers supporting its use at larger scale. Regarding growth performance, the fish fed the low marine protein diet only differed approximately 40 g from fish fed conventional diet at the end of the experiment. This difference can be reached in about 10–20 additional feeding days, which is considered as acceptable especially since fish fed the cost-effective low marine protein diet presented significant lower n-6 but higher n-3 content (i.e. lower n-6/n-3 ratio) when compared to conventional ones. In addition, the increase in the abundance of *Vibrio* and reduction of *Photobacterium* in the gut found in fish fed with the low marine protein diet appears to be opportunistic, but further investigation on sea bass health is needed. Indeed, no major health and welfare alterations were observed for fish fed the low marine protein diet and potential slight benefit related to humoral immunity was found. These results altogether support the use of such environmentally and economically sustainable diet in real farming conditions. Further research on newly cost-effective formulated aquafeeds containing fewer marine proteins and their impact on growth, health and welfare of marine fish will promote the European aquaculture environmentally sustainability.

### Supplementary Information

Supplementary Information.

## Data Availability

The data that support the findings of this study are available from the corresponding author upon request.
